# Progressive insights into fibrosarcoma diagnosis and treatment: leveraging fusion genes for advancements

**DOI:** 10.3389/fcell.2023.1284428

**Published:** 2023-10-18

**Authors:** Xiaodi Tang, Xin Hu, Yang Wen, Li Min

**Affiliations:** ^1^ Department of Orthopedic Surgery and Orthopedic Research Institute, West China Hospital, Sichuan University, Chengdu, Sichuan, China; ^2^ Model Worker and Craftsman Talent Innovation Workshop of Sichuan Province, Chengdu, Sichuan, China; ^3^ Department of Orthopedics, Zigong Fourth People’s Hospital, Zigong, China

**Keywords:** fusion genes, fibrosarcoma, generation mechanism, detection methods, targeted therapy

## Abstract

Fibrosarcoma, originating from fibroblast cells, represents a malignant neoplasm that can manifest across all genders and age groups. Fusion genes are notably prevalent within the landscape of human cancers, particularly within the subtypes of fibrosarcoma, where they exert substantial driving forces in tumorigenesis. Many fusion genes underlie the pathogenic mechanisms triggering the onset of this disease. Moreover, a close association emerges between the spectrum of fusion gene types and the phenotypic expression of fibrosarcoma, endowing fusion genes not only as promising diagnostic indicators for fibrosarcoma but also as pivotal foundations for its subcategorization. Concurrently, an increasing number of chimeric proteins encoded by fusion genes have been substantiated as specific targets for treating fibrosarcoma, consequently significantly enhancing patient prognoses. This review comprehensively delineates the mechanisms behind fusion gene formation in fibrosarcoma, the lineage of fusion genes, methodologies employed in detecting fusion genes within fibrosarcoma, and the prospects of targeted therapeutic interventions driven by fusion genes within the fibrosarcoma domain.

## 1 Introduction

Fibrosarcoma is a malignant neoplasm originating from mesenchymal tissues (Sbaraglia et al., 2021). Its proclivity for multi-organ affections is evident, with a conspicuous predilection for localization within the appendicular anatomy, particularly the proximal thigh. This pathologic entity predominantly targets the male demographic within the age bracket of 20–50 years, though instances of congenital presentation have been duly noted (Folpe, 2014; Augsburger et al., 2017). Contemporary approaches to diagnosing fibrosarcoma center on imaging and biopsy techniques. Nonetheless, both methods face challenges, including the high misdiagnosis rate associated with imaging and the limited detection efficacy of biopsies (Augsburger et al., 2017; Wang et al., 2018). Therapeutically, fibrosarcoma management revolves around surgical resection as the cornerstone, complemented by adjuvant chemotherapy, showcasing improved patient outcomes (Papagelopoulos et al., 2002). Consequently, the quest for precise early-stage diagnosis and effective personalized therapeutic strategies for fibrosarcoma emerges as pivotal for optimizing patient prognosis.

In recent times, an assemblage of fusion genes, notably exemplified by COL1A1-PDGFB, ETV6-NTRK3, and FUS-CREB3L2, has commanded scholarly attention. These fusion genes have incontrovertibly risen as pivotal agents catalyzing the genesis and advancement of fibrosarcoma (Kaye, 2009). Their predominant role lies in orchestrating these intricate processes through their associated proteins across a preponderance of fibrosarcoma instances. It's noteworthy that the majority of these fusion genes find involvement within the signal transduction pathways of cells, thus emerging as potential targets for fibrosarcoma therapy and forming a significant foundation for personalized clinical interventions (Simon et al., 2001; Peng et al., 2022). Moreover, the development of targeted therapeutic agents tailored to specific fusion genes has been realized. These interventions have demonstrated commendable efficacy, offering a transformative avenue in the therapeutic landscape for fibrosarcoma (Kérob et al., 2010; Bielack et al., 2019; Lapeña et al., 2022; Yang et al., 2022). Additionally, the accelerated and accurate detection of these fusion genes has become achievable, driven by the widespread integration of advanced methodologies such as fluorescence *in situ* hybridization (FISH) (Segura et al., 2011; Karanian et al., 2015; Solomon and Hechtman, 2019). This advancement holds the potential to cast a promising light on the future landscape, offering the prospect of considerably enhancing the precision of early-stage diagnosis for this enigmatic ailment.

Herein, this review aims to illuminate several recent breakthroughs in the realm of fusion genes implicated in fibrosarcoma. Furthermore, it endeavors to furnish a concise synthesis of innovative therapeutic methodologies poised to hold potential value in the forthcoming landscape of fibrosarcoma treatment.

## 2 Fusion gene formation mechanism in fibrosarcoma

A comprehensive understanding of the mechanisms governing the formation of fusion genes holds pivotal significance in the landscape of tumor therapeutics. This comprehension not only serves as a lodestar for tumor treatment strategies but also guides the development of precision-targeted therapies and individualized treatment regimens. For instance, the identification and subsequent targeted intervention of fusion proteins, exhibiting tumor-specific expression, offer the prospect of formulating tailored therapeutic protocols characterized by heightened efficacy, reduced adverse effects, and circumvention of drug resistance.

It is noteworthy that the mechanisms underpinning fusion gene formation predominantly encompass chromosomal rearrangements and aberrant transcriptional events (Annala et al., 2013; Mertens et al., 2015). In a substantial majority of fibrosarcoma cases, these aberrations engender novel fusion genes, thereby encoding distinct fusion proteins. Perturbation of transcription-associated genes through mutation instigates downstream alterations within pivotal signal transduction pathways, ultimately culminating in the perturbed cell proliferation, differentiation, and apoptosis dynamics that underlie the initiation and progression of fibrosarcoma (Albert et al., 2019; Forsythe et al., 2020).

Chromosomal rearrangement is a prevalent mechanism driving fusion gene generation in fibrosarcoma, encompassing deletions, tandem duplications, inversions, and translocations (Sandberg and Bridge, 2003; Edwards, 2010; Annala et al., 2013; Mertens et al., 2015; Heyer et al., 2019).

**FIGURE 1:**
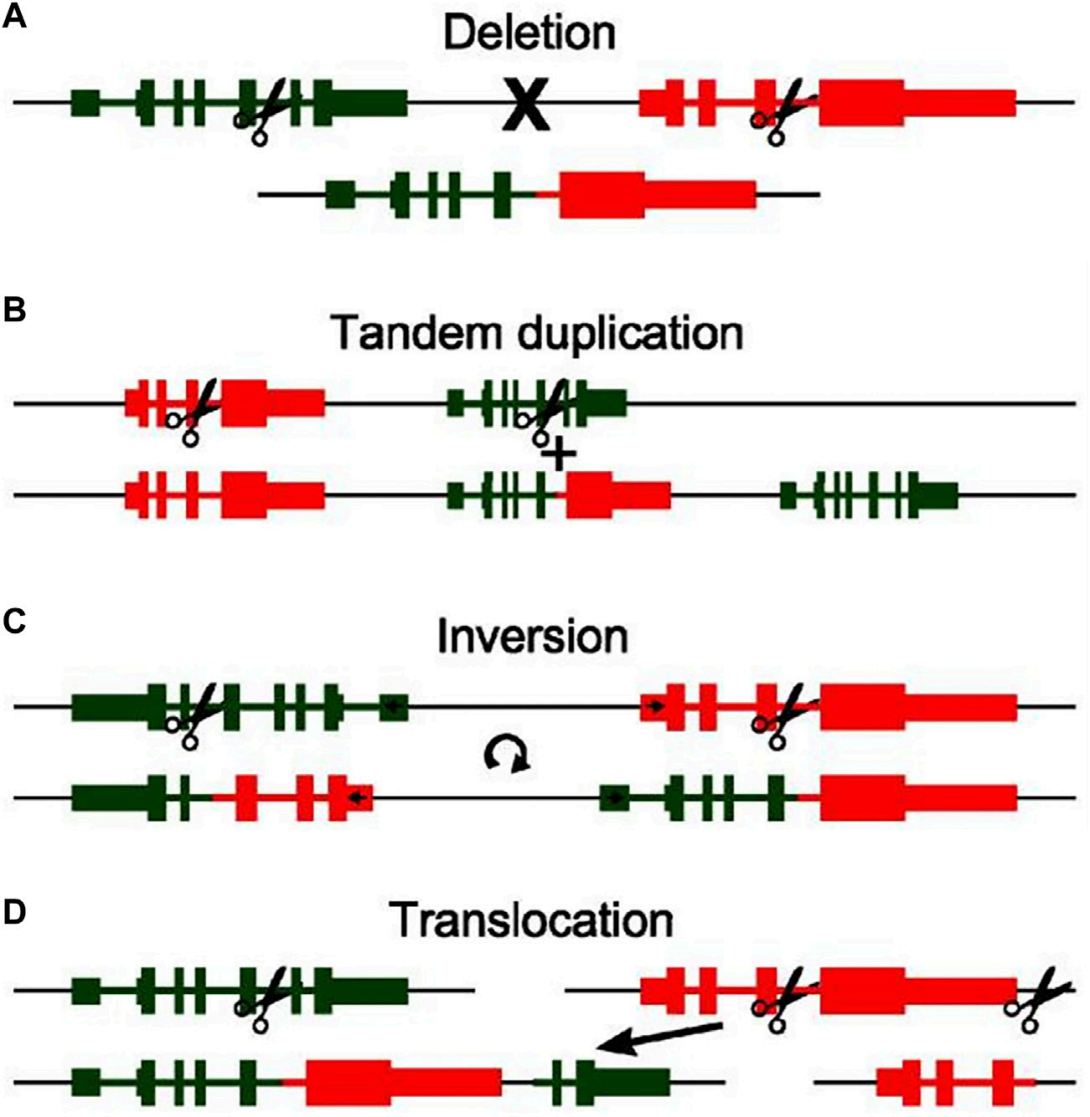
An illustration of the four basic types of chromosomal rearrangement and how they lead to the formation of fusion genes: Deletion **(A)**, Tandem duplication **(B)**, Inversion **(C)**, Translocation **(D)**. The original genomic layout is shown at the top, layout after rearrangement is shown at the bottom. Scissors indicate genomic breakpoints. A discontinuity in the black line indicates separate chromosomes (Reprinted with permission from Annala et al. (2013) Copyright ^©^ 2013, Cancer Lett, (Annala et al., 2013).

Deletion refers to the removal of a DNA fragment on the same strand (Figure 1A). The LMNA-NTRK1 fusion in Congenital fibrosarcoma is an example of a fusion that results from a 743-kb deletion on chromosome 1q. Increased phosphorylation levels of the fusion protein upon expression of lmna-ntrk1 fusion construct in congenital fibrosarcoma cells provide a novel avenue for treatment in congenital fibrosarcoma patients (Wiesner et al., 2014).

Interestingly, fusion genes can originate from tandem duplications, which characterized by the replication of a genomic region one or more times, resulting in adjacent positioning of the duplicated segments with the original locus (Figure 1B). When amplicon breakpoints are proximal to existing genes, the outcome can be the formation of fusion genes at the junction of the duplicated and original regions. This intricate process yields a multitude of fusion genes. For instance, within Dermatofibrosarcoma protuberans, exemplars like COL6A3-PDGFD and EMILIN2-PDGFD underscore fusion genes that materialize as a consequence of tandem duplication events (Peng et al., 2022). Furthermore, DNA repair, cell cycle, mitogen-activated protein kinase, phosphatidylinositol 3-kinase, and Janus kinase signaling pathways and transcription activation are crucial pathways implicated in the genetic alterations. These may aid in identifying potential diagnostic and therapeutic targets for DFSP.

Moreover, fusion genes can sporadically arise due to inversion events where segments of chromosomes undergo a reversal in orientation (Figure 1C). An illustrative example is the CSPG2-PTK2B fusion gene detected in Dermatofibrosarcoma protuberans, originating from a paracentric inversion spanning a substantial 71-Mb fragment between chromosomal regions 5q14.2 and 5q33.2 (Bianchini et al., 2008). Consequently, the presence of a fusion gene involving two genes located on opposing strands of the chromosome serves as an indicator of a potential inversion event (Annala et al., 2013). A distinctive attribute of this fusion type is the concurrent formation of mutually fused genes at both ends of the inverted segment (Ciampi et al., 2005).

In addition to the aforementioned chromosomal rearrangement mechanisms involving genes on the same chromosome, many fusion genes also result from genes located on different chromosomes. These fusions are often a consequence of various translocation events, including the transfer of small genomic fragments or the exchange of entire chromosomal arms (Figure 1D). For instance, the COL1A1-PDGFB fusion arises from a reciprocal translocation between chromosomal regions 17q and 22q (Simon et al., 1997). Although more intricate rearrangements are observed in fibrosarcoma, they are less common in practice (Lawson et al., 2011). Given that translocation necessitates involvement of at least two chromosomes, this chromosomal rearrangement approach appears more detectable in clinical practice compared to the aforementioned three methods. This might potentially contribute to early diagnosis for patients (Mertens et al., 2015).

## 3 Fusion genes in typical fibrosarcoma

### 3.1 Dermatofibrosarcoma protuberans (DFSP)

Dermatofibrosarcoma protuberans (DFSP) stands as a discernible entity within the realm of soft tissue sarcomas, characterized by its low-grade demeanor and local invasiveness. Emerging from the dermal or superficial subcutaneous layers of young to middle-aged adults, this neoplasm initially found its identity as keloid-like sarcomas before being designated as DFSP by Hoffman in 1925 (Iwasaki et al., 2019). Its prevalence encompasses around 5% of the entirety of soft tissue sarcomas and encompasses 18% of cutaneous soft tissue sarcomas, with a pronounced predilection for the Japanese population. Typically, DFSP manifests on the trunk, followed by occurrences on the head and neck. During its nascent growth phases, DFSP commonly manifests as polypoid protrusions or sclerotic plaques on the skin. Histologically, DFSPs are marked by the presence of moderately atypical yet homogenous spindle cells, meticulously arrayed in a storiform whorled configuration, intricately infiltrating the neighboring subcutaneous adipose tissue. The heterogeneity of DFSP is showcased through a multitude of histological subtypes, encompassing pigmented (Bednar tumor), myxoid, myoid, granular cell, sclerotic, atrophic, and fibrosarcomatous DFSPs, alongside giant cell fibroblastoma (Brenner et al., 1975; Llombart et al., 2013). Noteworthy patterns suggest that patients exhibiting advanced age, male gender, African ancestry, and specific anatomic tumor localization tend to exhibit suboptimal treatment outcomes (Criscito et al., 2016).

**FIGURE 2:**
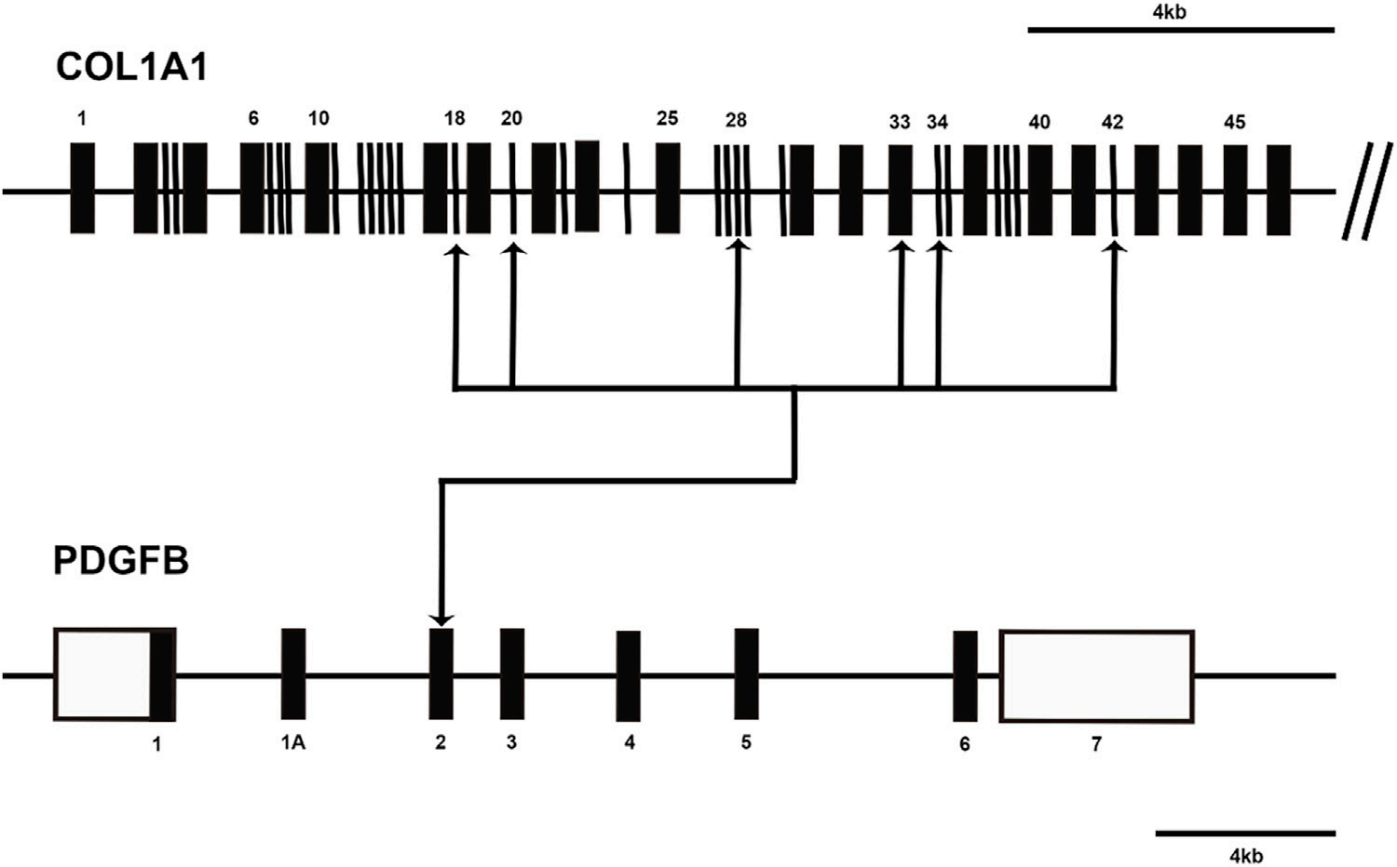
Diagram shows the COL1A1-PDGFB fusion transcripts in dermatofibrosarcoma protuberans (DFSP). The approximate locations of breakpoints within the COL1A1 gene and PDGFB gene are indicated by arrows. Open boxes represent untranslated regions.

In 1997, Simon et al. were pioneers in postulating the presence of a fusion between the platelet-derived growth factor beta chain (PDGFB) and collagen alpha 1 (COL1A1) genes in dermatofibrosarcoma protuberance (DFSP) and giant cell fibroblastoma (GCF). Their groundbreaking work illuminated the potential role of the COL1A1/PDGFB rearrangement in generating a growth factor endowed with cellular transforming activity (Simon et al., 1997). Subsequent cytogenetic investigations revealed that the COL1A1-PDGFB fusion gene emerges due to the translocation t (17; 22) (q22; q13), with the intriguing presence of a supernumerary circular chromosome, more frequently originating from translocation r (17; 22), observed in DFSP (Sandberg and Bridge, 2003). Delving into the cytogenetics of DFSP, scholars have mapped a diverse spectrum of breakpoints for COL1A1-PDGFB fusion genes. Among these, exon 2 of the PDGFB gene consistently participates in the fusion, while the COL1A1 gene presents multiple potential exons for fusion involvement, including exons 18, 20, 28, 33, 34, 42, and 44 (Maire et al., 2002; Saeki et al., 2003a; Saeki et al., 2003b; Saeki et al., 2005; Llombart et al., 2009; Otsuka-Maeda et al., 2020) (Figure 2). However, a subsequent study in 2009 revealed no discernible connection between distinct COL1A1 gene breakpoints and specific DFSP tissue types or clinical variables. Notably, the study pinpointed COL1A1 gene breakpoints primarily situated within the posterior half (exons 25–48) (Llombart et al., 2009). More recently, the detection of COL1A1-PDGFB fusions has garnered significant attention, being identified in 172 cases (91.4%) (Lee et al., 2022). This prevailing observation suggests the diagnostic potential of identifying COL1A1-PDGFB fusions as a valuable tool in the recognition of DFSP.

Within the domain of DFSP, a panorama of novel fusion genes and translocations has emerged, as summarized in Table 1. Prior cytogenetic investigations have shed light on potential translocation occurrences encompassing t (2; 17), t (9; 22) (q32; q12.2), t (X; 7), and t (5; 8) (Sinovic and Bridge, 1994; Craver et al., 1995; Sonobe et al., 1999; Bianchini et al., 2008). Recently, RNA sequencing has unveiled the discovery of four fresh fusion gene variants originating from DFSP (COL1A2-PDGFB, COL6A3-PDGFD, EMILIN2-PDGFD, SLC2A5-BTBD7) (Nakamura et al., 2015; Dadone-Montaudié et al., 2018; Dickson et al., 2018; Lee et al., 2022; Peng et al., 2022). Intriguingly, PDGFD rearrangement mirrors the classical PDGFB rearrangement in clinical, morphological, and molecular aspects, while the functional resemblance between the conventional COL1A1-PDGFB and alternative COL6A3-PDGFD or EMILIN2-PDGFD transcription products incites an oncogenic autocrine loop involving PDGFRB signaling (Dadone-Montaudié et al., 2018). An innovative translocation, t (1; 14), recently delineated in DFSP, engenders a fusion between the SLC2A5 and BTBD7 genes (Peng et al., 2022). Though the precise mechanistic underpinnings of the SLC2A5-BTBD7 fusion remain elusive, it introduces a novel potential target for diagnostic and therapeutic interventions in this condition.

**TABLE 1:** Novel chromosomal translocations or fusion genes in fibrosarcoma.

Tumor type	Translocation	Fusion gene		Reference
DFSP	t (2; 11) (q37.3; q22.3)	COL6A3-PDGF		Dickson et al. (2018)
	t (17; 22) (q33; q25)	COL1A2-PDGF		Nakamura et al. (2015)
	t (18; 11) (p11.32; q22.3)	EMILIN2-PDGFD		Dadone-Montaudié et al. (2018), Lee et al. (2022)
	t (1; 14)	SLC2A5-BTBD		Peng et al. (2022)
	t (5; 8)	CSPG2-PTK2B		Bianchini et al. (2008)
	t (2; 17)	Not mentioned		Sinovic and Bridge (1994)
	t (9; 22) (q32; q12.2)	Not mentioned		Sonobe et al. (1999)
	t (X; 7)	Not mentioned		Craver et al. (1995)
CFS/IFS	t (2; 15) (2p21; 15q25)	EML4-NTRK		Tannenbaum-Dvir et al. (2015)
	Not mentioned	LMNA-NTRK		Wong et al. (2016)
	Not mentioned	PHIP-BRAF		Boulouadnine et al. (2022)
LGFMS	Not mentioned	FUS-CREB3L1		Guillou et al. (2007)
	Not mentioned	EWSR1-CREB3L1		Lau et al. (2013)

### 3.2 Congenital (or infantile) fibrosarcoma (CFS/IFS)

Congenital fibrosarcoma, or infantile fibrosarcoma, primarily affects children aged 1 year or younger. Arising from fibroblastic and myofibroblastic origins, this tumor type is characterized by spindle cell morphology and demonstrates a low-grade malignancy with limited propensity for metastasis (Fisher, 1996). While sharing histological similarities with adult fibrosarcoma, congenital fibrosarcoma carries a more favorable prognosis. It predominantly emerges in the soft tissues of the extremities, trunk, head, and neck.

**FIGURE 3:**
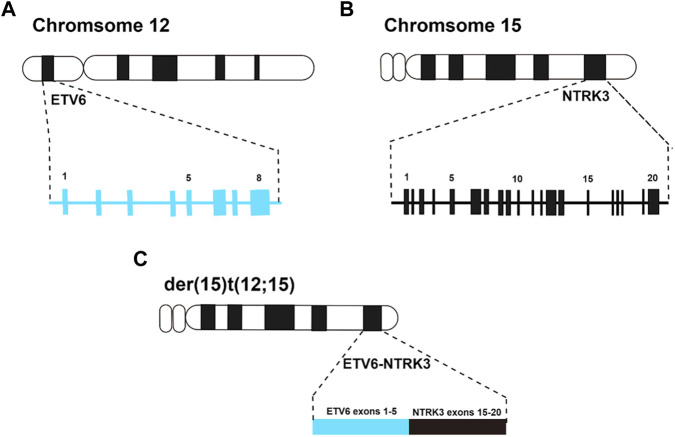
**(A)**, ideogram of chromosome 12 with the position of the ETV6 gene, and exons are indicated by numbers above the blue boxes; **(B)**, ideogram of chromosome 15 with the position of the NTRK3 gene, and exons are indicated by numbers above the black boxes; **(C)**, ideogram of der (15)t (12; 15) with the position of the ETV6-NTRK3 fusion gene, and the exons arrangement after fusion of ETV6 gene and NTRK3 gene.

Around 20 years ago, Knezevich et al. identified a novel fusion gene, ETV6-NTRK3, in congenital fibrosarcoma (CFS) (Knezevich et al., 1998). This landmark discovery not only implicated the NTRK receptor family in human tumorigenesis for the first time but also introduced the concept of ETV6 gene fusion within solid tumors. Subsequent investigations suggested that further rearrangements involving der (15)t (12; 15) or der (12)t (12; 15) in CFS might lead to a suppression of ETV6-NTRK3 molecular expression, potentially indicating the relatively benign nature of the tumor (Punnett et al., 2000) (Figure 3).

However, it is important to note that the classical ETV6-NTRK3 fusion gene is not universally present in all CFS patients. In recent years, several studies have uncovered additional fusion genes in CFS patients (as shown in Table 1). Notably, Tannenbaum-Dvir et al. reported a relapsed CFS case with an EML4-NTRK3 neo-fusion due to a t (2; 15) (2p21; 15q25) translocation. Interestingly, this patient responded better to chemotherapy and radiotherapy, challenging the notion that EML4-NTRK3 fusion invariably indicates a poor CFS prognosis (Tannenbaum-Dvir et al., 2015). In a similar vein, a 2016 study identified an ETV6-NTRK3-negative CFS case with an LMNA-NTRK1 gene fusion. This case exhibited near-complete responsiveness to crizotinib treatment (Wong et al., 2016). Furthermore, Boulouadnine et al. recently reported a CFS case with a novel PHIP-BRAF fusion gene, proposing it as a potential target for trametinib therapy (Boulouadnine et al., 2022). These instances of CFS with rare fusion genes not only expand our understanding but also introduce new therapeutic avenues for managing CFS patients.

### 3.3 Low-grade fibromyxoid sarcoma (LGFMS) and sclerosing epithelioid fibrosarcoma (SEF)

Low-grade fibromyxoid sarcoma (LGFMS) and sclerosing epithelioid fibrosarcoma (SEF) were initially delineated by Evans and Meis-Kindblom, respectively (Devaney et al., 1990; Meis-Kindblom et al., 1995). These entities, now recognized as two variants of fibrosarcoma, exhibit a strong association due to considerable morphological and genetic data.

LGFMS, a rare soft tissue tumor, primarily affects young adults while also impacting around 20% of cases in children (Evans, 1993; Guillou et al., 2007; Evans, 2011). Commonly arising in the deep soft tissues of extremities, LGFMS can emerge in diverse locations, including viscera (Laurini et al., 2011). Metastasis in LGFMS, with an approximate rate of 15%, typically transpires late (over 20 years post-diagnosis), necessitating prolonged patient follow-up (Laurini et al., 2011). Immunohistochemistry reveals fibroblast immunophenotype and MUC4 glycoprotein expression, a sensitive LGFMS marker (Doyle et al., 2011). Genetic evidence firmly confirms LGFMS as a solid tumor. Notably, t (7; 16) reciprocal translocations with rosette-like collagen loops were detected in LGFMS (Bejarano et al., 2000; Reid et al., 2003). This translocation fuses FUS and CREB3L2, with FUS-CREB3L2 detected in around 95% of LGFMS cases, and a variant FUS-CREB3L1 in less than 5% (Storlazzi et al., 2003; Lau et al., 2013). Coincidentally, these fusion genes are also present in SEF.

SEF, distinct as a fibrosarcoma variant, features collagen-rich stroma interspersed with epithelioid tumor cells, commonly presenting as a deep mass in extremities (Meis-Kindblom et al., 1995; Antonescu et al., 2001; Guillou et al., 2007; Evans, 2011). More aggressive than LGFMS, SEF exhibits higher metastasis rates and shorter survival (Warmke and Meis, 2021). Immunohistochemically, SEF typically shares marker expression with LGFMS. Genetically, SEF subpopulations, especially those resembling LGFMS, often bear FUS-CREB3L2 fusion via t (7; 16) (q34; p11)) (Guillou et al., 2007; Rekhi et al., 2011; Patterson et al., 2017). Conversely, “pure” SEFs may harbor EWSR1-CREB3L1 fusions via t (11; 22) (p11; q12) (Wang et al., 2012). Furthermore, recent investigations have unveiled novel fusion genes in SEFs (as outlined in Table 1). Notably, in 2017, Barbara et al. reported an intraperitoneal SEF case featuring an innovative EWSR1-CREB3L3 fusion identified through molecular genetic testing (Dewaele et al., 2017). Subsequent research highlighted the role of this fusion in aberrantly upregulating the PI3K/mTOR signaling pathway (Shenoy et al., 2019). In 2020, Khaled and Adham identified a mixed SEF/LGFMS case harboring a remarkably rare HEY1-NCOA2 fusion gene (Murshed and Ammar, 2020). Almost concurrently, another study disclosed a subset of MUC4-negative SEFs showcasing a YAP1-KMT2A gene rearrangement (Kao et al., 2020). Intriguingly, over half of the MUC4-negative SEFs exhibited this YAP1-KMT2A gene fusion. The exact mechanism behind this remains enigmatic, though it's likely that the complex and unbalanced chromosomal rearrangements underpinning YAP1-KMT2A contribute to the low detection rate of this fusion gene via FISH.

In summary, fusion genes play a pivotal role in various fibrosarcoma subtypes, profoundly influencing tumorigenesis. Detecting these fusion genes is imperative for precise diagnosis and treatment. Diverse assays are employed for fusion gene detection, each with pros and cons.

## 4 Detection of fibrosarcoma-associated fusion gene

The accurate detection of fibrosarcoma-associated fusion genes is a prerequisite for diagnosis. Currently, various experimental techniques are employed for clinical assessment of these fusion genes, encompassing fluorescence *in situ* hybridization (FISH), reverse transcription-polymerase chain reaction (RT-PCR), Pan-Trk immunohistochemistry (IHC), next-generation sequencing (NGS), among others. Each method presents distinct attributes, benefits, and constraints (summarized in Table 2).

**TABLE 2:** Features, advantages, and limitations of various methods.

	IHC	FISH	RT-PCR	DNA-based NGS	RNA-based NGS	DNA/RNA hybrid sequencing assays
Sensitivity	High	High	Variable	Variable	Very high	Very high
Specificity	High	High	Variable	Variable	Very high	Very high
Material	At least 1 slide	At least 3 slides	1 μg of RNA	250 ng of DNA.	200 ng of RNA.	10–40 ng of RNA
Time	1 day	1–3 days	1 week	2–4 weeks	2–4 weeks	2–4 weeks
Cost	Low	Low	Low	High	High	Very High

### 4.1 Fluorescence *in situ* hybridization (FISH)

The Fluorescence *in situ* hybridization (FISH) technique involves fluorescently labeled single-stranded DNA hybridizing with complementary DNA in a specimen. This technique highlights specific genes by indicating changes in fluorescent signal number and position. The interaction and quantity of signals are revealed using one or more DNA probes labeled with fluorescent molecules. Two primary approaches are employed for fusion identification: fusion probes, requiring knowledge of both partner genes, and break-apart probes targeting segments of a gene to identify intervening breaks caused by fusion events (Weiss and Funari, 2021).

**FIGURE 4:**
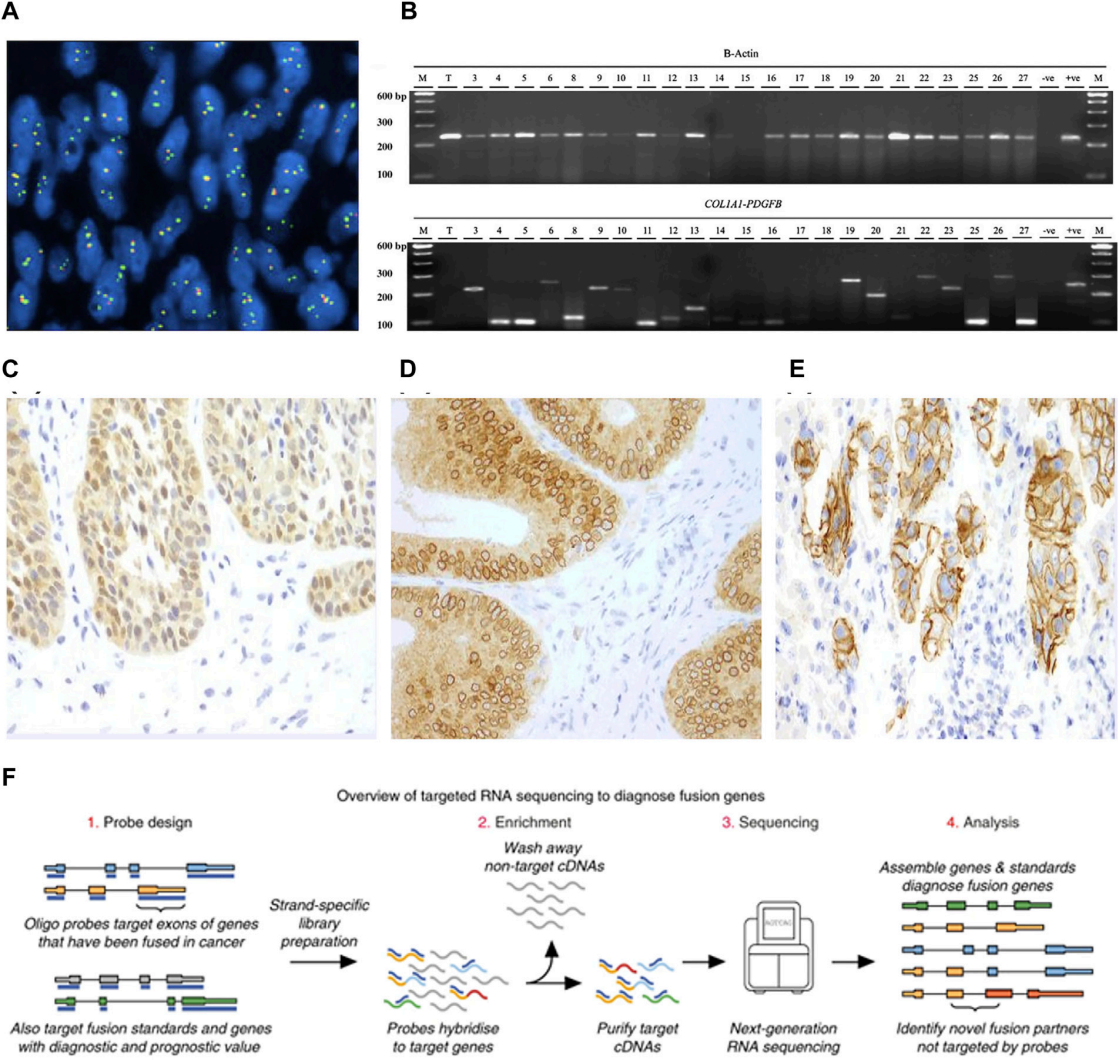
**(A)** FISH identification of PDGFB rearrangement. **(B)** RT-PCR analysis to diagnose COL1A1-PDGFB fusion [Reprinted with permission from Patel et al. (2008) Copyright ^©^ 2019, Ann Oncol, Bielack S. S, et al.]. **(C)** A nuclear and cytoplasmic staining pattern is seen in this case of secretory carcinoma of the salivary gland with the canonical ETV6-NTRK3 fusion. **(D)** Colonic adenocarcinoma with an LMNA-NTRK1 fusions exhibits a cytoplasmic and perinuclear staining pattern. **(E)** A membranous staining pattern is seen in this case of intrahepatic cholangiocarcinoma with a PLEKHA6-NTRK1 fusion [Reprinted with permission from Solomon et al. (2019) Copyright ^©^ 2019, Ann Oncol, J. P. Solomon, et al.] **(F)** Schematic of targeted RNAseq process [Reprinted with permission from Heyer et al. (2019) Copyright ^©^ 2019, Erin E, et al.].

For diagnosing DPFS, FISH is employed with a positive criterion being the identification of a COL1A1-PDGFB fusion gene or PDGFB rearrangement in at least 10% of tumor cells (Iwasaki et al., 2019) (Figure 4A). FISH’s capability to detect variant PDGFB rearrangements enhances its clinical sensitivity in diagnosing PDGFB fusion genes in DPFS (Patel et al., 2008). In detecting ETV6-NTRK3 fusions, a widely used commercial probe is the isolated ETV6 probe, effectively confirming ETV6-NTRK3 rearrangements in infantile fibrosarcomas (Solomon and Hechtman, 2019). Positive FISH results manifest as split or isolated signals, with thresholds ranging from 5% to 15% of tumor cells (Solomon and Hechtman, 2019). Offering swift results and demanding minimal tissue, FISH has become the prevalent molecular genetic technique for diagnosing fusion genes in DPFS, LGFMS, and SEF.

### 4.2 RT-PCR

RT-PCR, a precision RNA-level technique, employs specific primers to uncover oncogenic fusions in fibrosarcoma (Solomon et al., 2019; Schmitt et al., 2021). It amplifies target DNA products through denaturation, annealing, and extension cycles, spotlighting fusion transcripts (Weiss and Funari, 2021) (Figure 4B). Widely used like FISH, RT-PCR offers a quick 3–7-day turnaround and cost-effectiveness. It's pivotal for identifying fusion genes such as COL1A1-PDGFB in DFSP and NTRK in CFS, while also quantifying tumor burden and aiding post-treatment monitoring. However, fusion gene complexity limits its clinical utility due to variable constituents and breakpoints.

### 4.3 Pan-Trk IHC

IHC, a potent technique leveraging antigen-antibody binding, pinpoints specific antigens in cells and tissues under light microscopy (Magaki et al., 2019). Yet, traditional IHC’s limitation is one marker per tissue section (Tan et al., 2020), missing vital diagnostic insights. To address this, advanced IHC-based methods have emerged, meeting heightened demands. PAN-Trk IHC, for instance, is a sensitive, specific, and resource-efficient technique for NTRK fusion identification in CFS (Hechtman et al., 2017) (Figures 4C–E). This serves as an adjunct or alternative to nucleic acid assays, enhancing diagnostic power. IHC’s simplicity, affordability, swiftness, sensitivity, and specificity make it valuable not only for fibrosarcoma fusion gene detection but also prognosis evaluation (Yong et al., 2014; Chen and Lin, 2015; Solomon et al., 2019).

### 4.4 NGS

Sanger sequencing, a pioneering technique, involves DNA polymerase extending primers binding to the target sequence. In contrast, Next-Generation Sequencing (NGS) offers vast genetic insights at reduced cost and time, with high yield and resolution. NGS’s prowess lies in parallel sequencing of numerous DNA molecules, detecting diverse cancer mutation types like single nucleotide variants, insertions, deletions, copy number variants, structural changes, and gene fusions (Weiss and Funari, 2021). Yet, NGS can be pricier and slower than single-assay methods, demanding complex protocols and about 2 weeks for results. While both DNA-based and RNA-based NGS exist, the latter is the gold standard for fibrosarcoma fusion gene identification (Solomon et al., 2019; Pfarr et al., 2020). RNA-based NGS excels as fusion transcripts are highly expressed, enabling detection even in low-tumor-purity samples, offering heightened sensitivity and specificity in fibrosarcoma fusion gene identification (Figure 4F).

## 5 Targeted therapy based on fibrosarcoma-associated fusion genes

Targeted therapy based on Fibrosarcoma-Associated Fusion Genes holds promising therapeutic potential. Precise intervention targeting the fusion gene products or their associated signaling pathways can effectively suppress tumor growth and progression while minimizing impact on normal cells. This therapeutic approach holds prospects for personalized treatment, offering the potential for more effective and less adverse treatment options. However, the current landscape presents limited targeted therapeutics for fibrosarcoma fusion genes, necessitating in-depth exploration of the mechanistic underpinnings of fusion genes and the efficacy and safety of therapeutic agents. Currently, typical targeted therapies based on Fibrosarcoma-Associated Fusion Genes primarily involve COL1A1-PDGFB and ETV6-NTRK3 (summarized in Table 3).

**TABLE 3:** The ongoing trials with targeted therapy against fibrosarcoma.

Tumor type	Targeted therapy	Reference
DFSP	Imatinib	Gooskens et al. (2010), Kérob et al. (2010)
CFS/IFS	Larotrectinib	Laetsch et al. (2018), Bielack et al. (2019), Yang et al. (2022)
	Entrectinib	Pollack et al. (2021)
	Crizotinib	Bender et al. (2019)
	PKC412	Chi et al. (2012)
	LOXO-101	Nagasubramanian et al. (2016)
LGFMS	Not mentioned	Not mentioned

### 5.1 COL1A1-PDGFB-based targeted therapy

**FIGURE 5:**
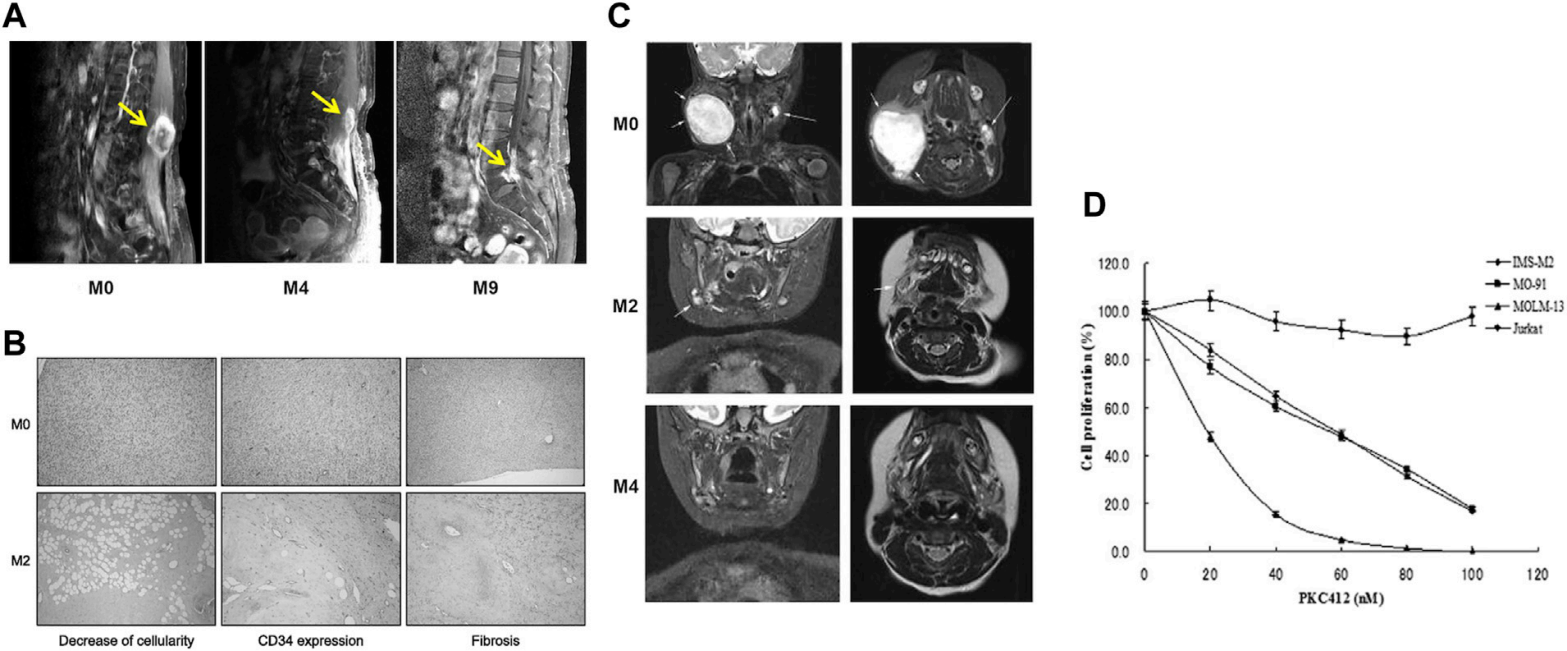
**(A)** A 46-year old women with DFSP at paravertebral site at the level of L3–4. Respectively before imatinib therapy, the use of imatinib therapy after 4 months, the use of imatinib therapy after 9 months of the lumbar spine MRI results [Reprinted with permission from Hong et al. (2013) Copyright ^©^ 2013, Hong, et al.]. **(B)** Decrease of cell density and CD34 expression as well as induction of hyalinic fibrosis in one patient’s lesion after 2 months therapy with IM. **(C)** Coronal and planar magnetic resonance imaging of a patient with IFS before starting treatment with larotrectinib, 2 months after treatment, and 4 months after treatment [Reprinted with permission from Bielack et al. (2019) Copyright ^©^ 2019, Ann Oncol, Bielack S. S, et al.]. **(D)** the anti-proliferation effect of PKC412 in cell lines harboring EN. Cell lines including IMS-M2, M0–91, MOLM-13 and Jurkat at a density of 1 × 105cells/mL were treated with 10, 20, 40, 60, 80, 100 nM PKC412 or DMSO alone (0 nM PKC412) as control for 72 h. The number of alive cells was counted after trypan blue exclusion test. Results were calculated as the percentage of the control values.

Recent genomic and molecular investigations have illuminated the prevalence of the COL1A1-PDGFB fusion gene in DFSP patients, highlighting its pivotal role in DFSP cells and presenting a novel avenue for clinical intervention. Although Mohs micrographic surgery remains the primary treatment for DFSP, its efficacy is mitigated by a notable recurrence rate (Ugurel et al., 2019). Moreover, conventional chemotherapy yields limited response in DFSP, contributing to its challenging prognosis. Encouragingly, strides in genomic and molecular research have unearthed potential candidates for molecularly targeted therapies. Several molecularly targeted approaches have been explored for unresectable DFSP, with imatinib mesylate, a tyrosine kinase inhibitor, emerging as a promising option. Given that DFSP patients harbor the COL1A1-PDGFB fusion gene, studies have unveiled the critical role of tyrosine kinase in mediating the action of COL1A1-PDGFB protein within DFSP cells. Consequently, almost two decades ago, investigations revealed that the sustained stimulatory impact of PDGFB receptors on DFSP cells could be counteracted by tyrosine kinase inhibitors, effectively impeding tumor progression (Breuninger et al., 2004). It wasn't until the past decade that imatinib gained recognition as a viable targeted therapy for DFSP, proving beneficial as a neoadjuvant treatment for unresectable or locally advanced tumors (Kérob et al., 2010; Hong et al., 2013) (Figures 5A, B). Additionally, fusion genes could potentially influence the evolution of DFSP and hinder regression by influencing DNA repair, cell cycle processes, and mitogen-activated protein kinase pathways (Peng et al., 2022). Ongoing endeavors are necessary to unearth novel therapeutic strategies targeting fusion proteins, aiming to enhance prognosis and bolster survival rates among DFSP patients.

### 5.2 ETV6-NTRK3-based targeted therapy

**FIGURE 6:**
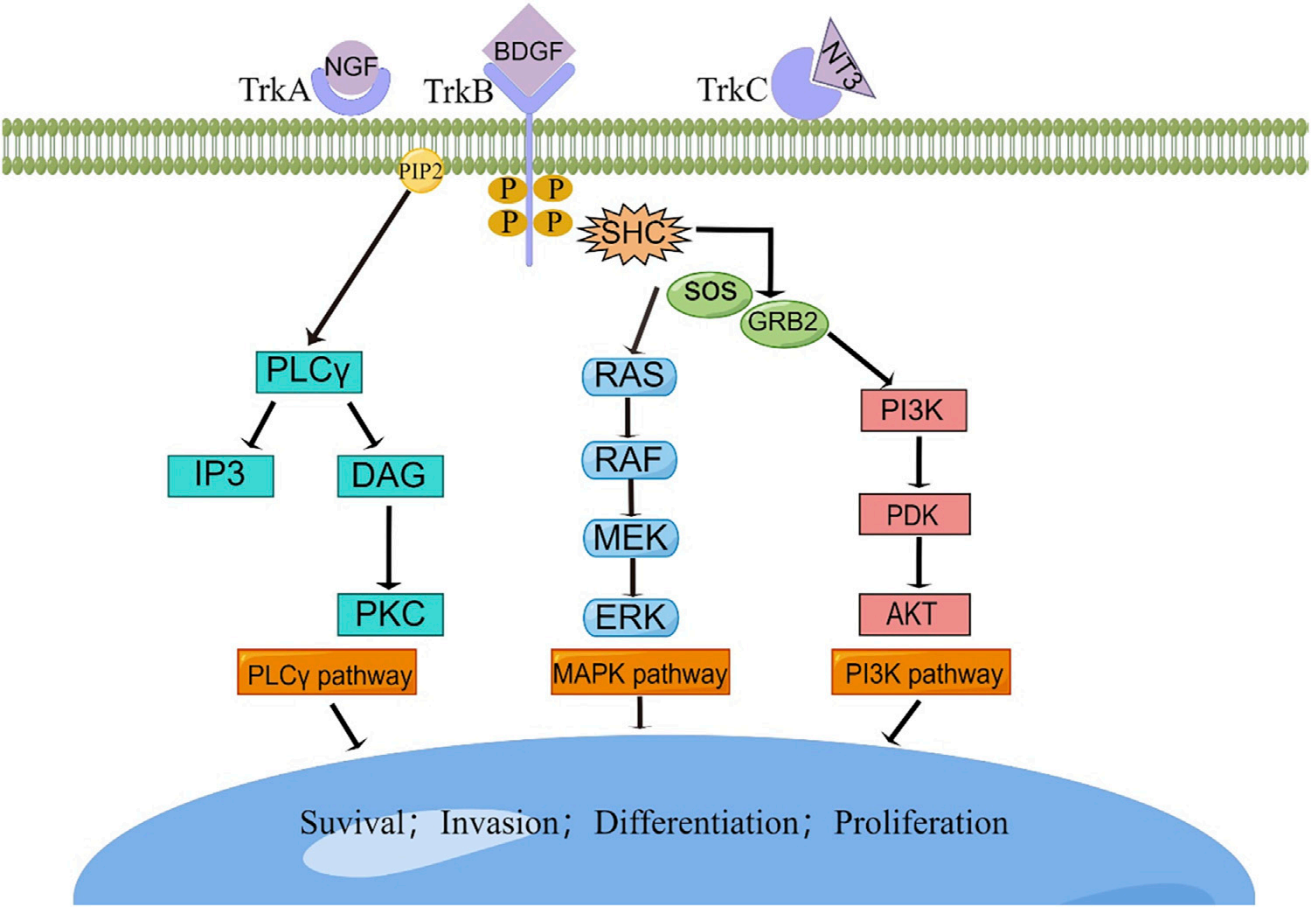
The TRK signaling pathways. Interaction between TRK and its cognate ligand will lead to downstream signal transduction, resulting in the activation of intracellular pathways responsible for cellular survival, invasion, differentiation, and proliferation. BDGF, brain-derived growth factor; DAG, diacylglycerol; ERK, extracellular signal-regulated kinase; GRB2, growth factor receptor-bound protein 2; IP3, inositol trisphosphate; MAPK, mitogen-activated protein kinase; MEK, mitogenactivated protein kinase; NGF, nerve growth factor; NTF-3, neurotrophin 3; PDK, phosphoinositide-dependent kinase; PI3K, phosphatidylinositol-4,5-bisphosphate 3-kinase; PIP2, phosphatidylinositol 4,5-bisphosphate; PKC, protein kinase C; PLC-γ, phospholipase C-γ; RAF, rapidly accelerated fibrosarcoma kinase; RAS, rat sarcoma kinase; SHC, Src homology 2 domain containing; SOS, sons of sevens.

While congenital fibrosarcoma (CFS) is a rare malignancy, its survival rates remain suboptimal. Surgical resection, despite its associated high recurrence rate, continues to be the gold standard treatment. In cases where radical resection is unfeasible, neoadjuvant chemotherapy, employing vincristine, actinomycin, and cyclophosphamide, has demonstrated efficacy and enables subsequent tumor resection (Ferrari et al., 2012). Notably, the prevalence of the ETV6-NTRK3 fusion in this tumor has prompted recent investigations into targeted therapies. The neurotrophic tyrosine receptor kinases (NTRK1, NTRK2, and NTRK3) encode tropomyosin-associated kinases A, B, and C (TrkA, TrkB, and TrkC)—receptor tyrosine kinases. These receptors share structural homology, featuring leucine-rich motifs, cysteine clusters, and immunoglobulin-like I-set domains within their extracellular domains (Klein et al., 1991). Ligand-binding regions are encompassed by the immunoglobulin-like domain. Neurotrophic factors, such as nerve growth factor, brain-derived growth factor, and neurotrophin factor 3/4, activate TrkA, TrkB, and TrkC, respectively. TRKs play pivotal roles in the physiological development and function of the peripheral and central nervous systems (Nakagawara, 2001; Arévalo and Wu, 2006). Ligand-receptor binding drives receptor dimerization and subsequent activation domain phosphorylation, initiating downstream intracellular signaling pathways that foster cell proliferation, differentiation, and survival, culminating in tumorigenesis (Kaplan et al., 1991) (Figure 6). Recent investigations have unveiled that small-molecule tyrosine kinase inhibitors, PKC412 and LOXO-101, exhibit promise in CFS treatment by suppressing TRK activation (Chi et al., 2012; Nagasubramanian et al., 2016) (Figures 5C, D). Novel TRK inhibitor Larotrectinib has yielded swift, enduring responses and a low incidence of adverse events. However, resistance may develop with prolonged usage. An ongoing prospective multicenter study aims to assess Larotrectinib’s safety and efficacy in the clinical setting (Yang et al., 2022). Collectively, these studies indicate that TRK inhibitors may offer innovative avenues for CFS treatment by targeting ETV6-NTRK3 activation inhibition.

Nonetheless, within the realm of fibrosarcoma fusion genes, a realm of unexplored possibilities lingers. This encompasses 1) the evolution of detection technologies to unearth additional distinctive fusion genes, thereby refining diagnostic acumen; 2) a holistic understanding of the oncogenic mechanisms, underpinning targeted therapeutic strategies with robust evidence; 3) a concerted endeavor in comprehensive preclinical and clinical trials, quintessential to validate the efficacy of fusion gene-directed interventions. Evidently, we stand at the cusp of an era marked by profound advancement in fusion gene detection and targeted therapeutic paradigms, poised to usher in bespoke treatments that elevate the quality of life and survival prospects for fibrosarcoma patients.

## 6 Conclusion

This review provides a comprehensive overview of the intricate mechanisms governing the origin of distinctive fusion genes across diverse fibrosarcoma subtypes, elucidating their detection modalities and pivotal roles in propelling fibrosarcoma progression. Notably, signature fusion genes unique to DFSP, CFS, and SEF/LGFMS have been identified, exerting their tumorigenic influence by modulating intricate cellular signaling cascades. Significantly, the emergence of targeted therapies aimed at these fusion genes is gaining momentum, offering a novel therapeutic avenue in the management of fibrosarcoma.
